# Mailed fecal testing and patient navigation versus usual care to improve rates of colorectal cancer screening and follow-up colonoscopy in rural Medicaid enrollees: a cluster-randomized controlled trial

**DOI:** 10.1186/s43058-022-00285-3

**Published:** 2022-04-13

**Authors:** Gloria D. Coronado, Michael C. Leo, Katrina Ramsey, Jennifer Coury, Amanda F. Petrik, Mary Patzel, Erin S. Kenzie, Jamie H. Thompson, Erik Brodt, Raj Mummadi, Nancy Elder, Melinda M. Davis

**Affiliations:** 1 Kaiser Permanente Center for Health Research, 3800 N. Interstate Ave, Portland, OR 97227 USA; 2 Oregon Rural Practice-Based Research Network, 3181 S.W. Sam Jackson Park Road, Mail code: L222, Portland, OR 97239-3098 USA; 3 OHSU Biostatistics and Design Program, 3181 S.W. Sam Jackson Park Road, Mail code: CB669, Portland, OR 97239-3098 USA; 4 OHSU Family Medicine, OHSU School of Medicine, 3181 S.W. Sam Jackson Park Road, Mail code: L222, Portland, OR 97239-3098 USA; 5 OHSU-PSU School of Public Health, 3181 S.W. Sam Jackson Park Road, Mail code: L222, Portland, OR 97239-3098 USA

**Keywords:** Medicaid, Colorectal cancer screening, Mailed FIT outreach, Fecal test, Fecal immunochemical test (FIT), Follow-up colonoscopy, Implementation science, Rural, Primary care

## Abstract

**Background:**

Screening reduces incidence and mortality from colorectal cancer (CRC), yet US screening rates are low, particularly among Medicaid enrollees in rural communities. We describe a two-phase project, SMARTER CRC, designed to achieve the National Cancer Institute Cancer Moonshot^SM^ objectives by reducing the burden of CRC on the US population. Specifically, SMARTER CRC aims to test the implementation, effectiveness, and maintenance of a mailed fecal test and patient navigation program to improve rates of CRC screening, follow-up colonoscopy, and referral to care in clinics serving rural Medicaid enrollees.

**Methods:**

Phase I activities in SMARTER CRC include a two-arm cluster-randomized controlled trial of a mailed fecal test and patient navigation program involving three Medicaid health plans and 30 rural primary care practices in Oregon and Idaho; the implementation of the program is supported by training and practice facilitation. Participating clinic units were randomized 1:1 into the intervention or usual care. The intervention combines (1) mailed fecal testing outreach supported by clinics, health plans, and vendors and (2) patient navigation for colonoscopy following an abnormal fecal test result. We will evaluate the effectiveness, implementation, and maintenance of the intervention and track adaptations to the intervention and to implementation strategies, using quantitative and qualitative methods. Our primary effectiveness outcome is receipt of any CRC screening within 6 months of enrollee identification. Our primary implementation outcome is health plan- and clinic-level rates of program delivery, by component (mailed FIT and patient navigation). Trial results will inform phase II activities to scale up the program through partnerships with health plans, primary care clinics, and regional and national organizations that serve rural primary care clinics; scale-up will include webinars, train-the-trainer workshops, and collaborative learning activities.

**Discussion:**

This study will test the implementation, effectiveness, and scale-up of a multi-component mailed fecal testing and patient navigation program to improve CRC screening rates in rural Medicaid enrollees. Our findings may inform approaches for adapting and scaling evidence-based approaches to promote CRC screening participation in underserved populations and settings.

**Trial registration:**

Registered at clinicaltrial.gov (NCT04890054) and at the NCI’s Clinical Trials Reporting Program (CTRP #: NCI-2021-01032) on May 11, 2021.

Contributions to the literature

- Our study will contribute new data on the effectiveness, implementation, and maintenance of a program of mailed fecal test outreach and patient navigation to improve screening rates in rural settings.
- We use a collaborative model that involves health plans, clinics, and vendors. Our findings will provide evidence for the scalability of this model across similar US settings.
- Our evaluation will track multi-level adaptations to the intervention and to implementation strategies using novel frameworks, contributing to scant prior research in this area.
- We will scale up the study to over 130 rural clinics and assess the program adoptions and adaptations (both planned and executed).

## Background

Colorectal cancer (CRC) is the third-leading cause of cancer death in the USA [1, 2]. CRC is 90% curable with timely detection and appropriate treatment of precancerous growths [3]. If not found until a patient is symptomatic, however, survival rates drop to 50% [4]. While CRC screening could prevent up to 60% of deaths from CRC, approximately 33% of US adults aged 50–75 (25 million people) are not currently up to date with CRC screening guidelines [5] [6]. Moreover, recent changes to the US Preventive Services Task Force guidelines, calling for the initiation of CRC screening at age 45 rather than 50, will result in over 20 million additional adults needing CRC screening services [7–9].

Rates of CRC screening are particularly low among adults in rural communities and among specific sub-populations within these settings, including Medicaid enrollees [3, 10–12]. Rural areas cover 97% of the US land area and are home to about 60 million people [13]. CRC incidence and mortality are disproportionately high among residents of rural regions compared to residents of urban regions [11, 14]; these disparities are driven primarily by differences in adherence to screening guidelines [11, 15]. Medicaid enrollees are a key underserved group in rural areas. In 2019, Medicaid provided health insurance and access to preventive health services to 71 million people, including nearly 1 in 4 rural US residents under age 65 (24%) [16–18]. Medicaid members between the ages of 50 and 64 years have relatively low rates of CRC screening (54%) compared to commercially insured adults in the same age group (65%) [19, 20]. Moreover, administrative claims data from Oregon show that newly age-eligible Medicaid enrollees (i.e., those turning 50) are 33% less likely to initiate CRC screening compared to newly age-eligible commercially insured adults [21]. Medicaid members also have less favorable CRC outcomes on average than commercially insured adults [22, 23].

Interventions are needed to address disparities in CRC screening and follow-up in rural Medicaid enrollees. Fecal immunochemical testing (FIT) is a convenient at-home screening strategy recommended by the US Preventive Services Task Force; adults with an abnormal FIT result must obtain a follow-up colonoscopy to find and remove polyps or find cancers at early stages. Several systematic reviews have highlighted the success of mailed FIT outreach as a strategy to improve rates of CRC screening, demonstrating greater CRC screening improvements than with other strategies [24–27]. Patient navigation has also demonstrated promise for raising rates of follow-up colonoscopy completion among individuals with abnormal FIT results [28–30]. Findings from microsimulation modeling conducted by our team show that combining mailed FIT outreach with patient navigation is a cost-effective strategy that can lead to CRC screening rates within Medicaid enrollees that exceed national CRC screening targets over 5 years of implementation [31].

Our study, Screening More patients for CRC through Adapting and Refining Targeted Evidence-based Interventions in Rural settings (SMARTER CRC), evaluates the implementation of a targeted, multi-level program that incorporates tailored mailed FIT outreach and patient navigation to address CRC disparities in rural Medicaid populations. Through the course of a large-scale pragmatic trial plus a scale-up study, we anticipate working with more than 20 regional and national organizations to facilitate the program’s implementation in an estimated 160 rural primary care clinics (serving over 21,000 rural Medicaid patients aged 50–75). We will assess adaptations and drivers of program success at the patient, clinic, health plan, vendor, and policy levels. This study fills key evidence and implementation gaps and will support President Biden’s Cancer Moonshot^SM^ objectives by developing a model for how to rapidly adapt and scale-up multi-level interventions through clinic-health plan-vendor partnerships to reduce the burden of CRC on the US population.

## Methods

### Study overview

SMARTER CRC is being conducted as part of the NCI-funded consortium, the Accelerating Colorectal Cancer Screening and Follow-up through Implementation Science (ACCSIS) Program. The overall aim of ACCSIS is to conduct multi-site, coordinated, transdisciplinary research to evaluate and improve CRC screening processes using implementation science strategies. SMARTER CRC is a large-scale, parallel, cluster-randomized study involving three Medicaid health plans and 30 rural clinics, followed by a scale-up study involving 130 rural practices. A cluster-randomized design was chosen because it could minimize the potential for contamination (versus individual-randomized designs) and could minimize bias due to changes in secular trends (versus stepped-wedge designs). Clinic units were randomized either to implement mailed FIT outreach along with a tailored patient navigation program to promote timely follow-up for abnormal FIT or to continue providing usual care. The intervention program was tailored for rural Medicaid populations using Boot Camp Translation [32], with components delivered by the health plan, clinic, and a direct-mail vendor. We will evaluate effectiveness by comparing rates of any CRC screening in eligible enrollees in the intervention and usual care clinics. We will also assess adaptations made prior to, during, or following program implementation, as well as the program’s implementation, and second-year maintenance. The study is a collaborative partnership between the Oregon Health & Science University’s Oregon Rural Practice-based Research Network and the Kaiser Permanente Northwest Center for Health Research. Study activities build on prior mailed FIT and patient navigation implementation research conducted by our team [32–44], best practices identified at a Centers for Disease Control Mailed FIT Summit [45], rural cancer control recommendations by rural health experts [46], participatory implementation science principles [47], and formative research conducted by the Community Health Advocacy and Research Alliance [31, 48–50].

SMARTER CRC has obtained approval from the Oregon Health & Science University’s Institutional Review Board (protocol number: 20681), which has granted a waiver of informed consent because the study involves minimal risks to enrollees and could not practicably be conducted without the waiver. A ceding agreement was obtained from Kaiser Permanente Center for Health Research. The study will be monitored by the MPIs, following an established internal data safety monitoring plan.

### Setting description

The project team invited participation from eight health plans that serve rural counties in Oregon; three health plans agreed to participate (37.5%). All three health plans are Coordinated Care Organizations (CCOs), networks of heath care providers (physical, mental, and dental) who work with local communities to provide coordinated care to individuals covered on Medicaid. The three participating CCOs are Eastern Oregon CCO (EOCCO), Cascade Health Alliance (CHA), and PacificSource. PacificSource is organized into administrative entities that cover three distinct regions participating in SMARTER CRC: PacificSource-Central, PacificSource-Gorge, and PacificSource-Marion and Polk Counties. In 2021, these CCOs provided Medicaid and dual-eligible coverage to 296,047 people in Oregon (64,902 at EOCCO; 23,912 at CHA; and 207,233 at PacificSource). The 2019 Medicaid CRC screening rate was 51% for EOCCO and 52% for CHA, and the 2021 Medicaid CRC screening rate was 62% for PacificSource (62% for PacificSource-Central, 63% for PacificSource-Gorge, and 62% for PacificSource-Marion and Polk Counties).

All participating CCOs provide coverage with no out-of-pocket costs to Medicaid enrollees for FIT, colonoscopy, sigmoidoscopy, CT colonography, and follow-up colonoscopy after an abnormal test result in alignment with Oregon policy [51, 52]. For dual-eligible enrollees, health plans also cover multi-target stool DNA (mt-sDNA). CRC screening is covered for all CCO enrollees aged 50–75 years, and in 2021 coverage was expanded to include adults aged 45–49 years to align with the recent US Preventive Services Task Force guideline changes [53, 54].

Participating CCOs promote CRC screening in a variety of ways. In prior years, a subset of EOCCO clinics (4 clinics in 2017, 6 clinics in 2018, and 5 clinics in 2019) conducted a mailed FIT outreach program using the collaborative clinic-health plan-vendor model from the BeneFIT study (same model as the present study; the present study uses rural-adapted materials) [55–57]. Implementation support was provided to EOCCO clinics by our team at the Oregon Rural Practice-based Research Network. CHA has also engaged in previous mailed FIT outreach efforts, including a 2019 effort in which they directly mailed FITs to Medicaid enrollees and a 2020 effort in which they provided FITs to one large, affiliated clinic which mailed them to Medicaid enrollees. In 2018, PacificSource-Gorge operated a mailed FIT outreach program for Medicaid enrollees receiving care at three rural clinics as part of a pilot study led by our team. Since 2018, PacificSource has contracted with a vendor to run a system-wide mailed outreach program to all Medicare enrollees in the Gorge and Central Oregon (*n* = 6795 in 2021), and in 2019 expanded the program to include prior-year FIT-screened Medicaid enrollees (*n* = 239 in 2021). The SMARTER CRC intervention will be overlaid on these existing screening promotion efforts.

The project team (MPI, research associate, project manager, practice facilitator) collaborated with the participating CCOs to recruit affiliated clinics. To be eligible for participation in the study, clinics had to have at least 30 age-eligible Medicaid or dual-eligible enrollees, have a CRC screening rate of 60% or lower, and be in a geographic region designated as rural or frontier by the Oregon Office of Rural Health or have a Rural-Urban Commuting Area code of 4 or higher [58–60]. For EOCCO, CHA, and most PacificSource regions, we used clinic-level Medicaid claims data from 2019 for enrollees aged 50–75 to determine clinic eligibility. For clinics in one PacificSource region, 2019 claims data were unavailable; thus, we used claims data from 2021. We opted not to use data from 2020 because the care suspensions during the COVID pandemic led to temporary reductions in CRC screening rates. A total of 33 clinics (organized into 29 clinic units for randomization) were recruited between May 2020 and February 2021; each signed a participant agreement letter confirming their plans to participate in the study. Clinics generally offer opportunistic screening through in-clinic FIT distribution or referral to colonoscopy; some offer mt-sDNA to patients with Medicare or commercial insurance.

### Research aims

Our study has the following aims:

- Aim 1: Conduct a large-scale pragmatic study, using a two-arm cluster-randomized design, to assess the implementation, effectiveness, and maintenance of a direct mail and patient navigation CRC screening program in 30 rural primary care clinics (*n* ~ 3960 patients aged 50–64), using practice facilitation to support implementation. Using a mixed methods approach, identify patient-, clinic/health system-, and payer/policy/community-level factors that are associated with reach, effectiveness, implementation, and maintenance of the program, and assess program adaptations.
- Aim 2: Partner with regional and national organizations (*n* ~ 20) to scale up the program to clinics serving rural and underserved patients in high-priority geographic regions of the USA (*n* ~ 130 clinics; 17,000+ patients) using webinars, train-the-trainer workshops, and collaborative learning approaches. Assess trainings delivered, program adoption and adaptations, and determinants of dissemination success.

#### Aim 1: large-scale, cluster-randomized pragmatic study

**Fig. 1:**
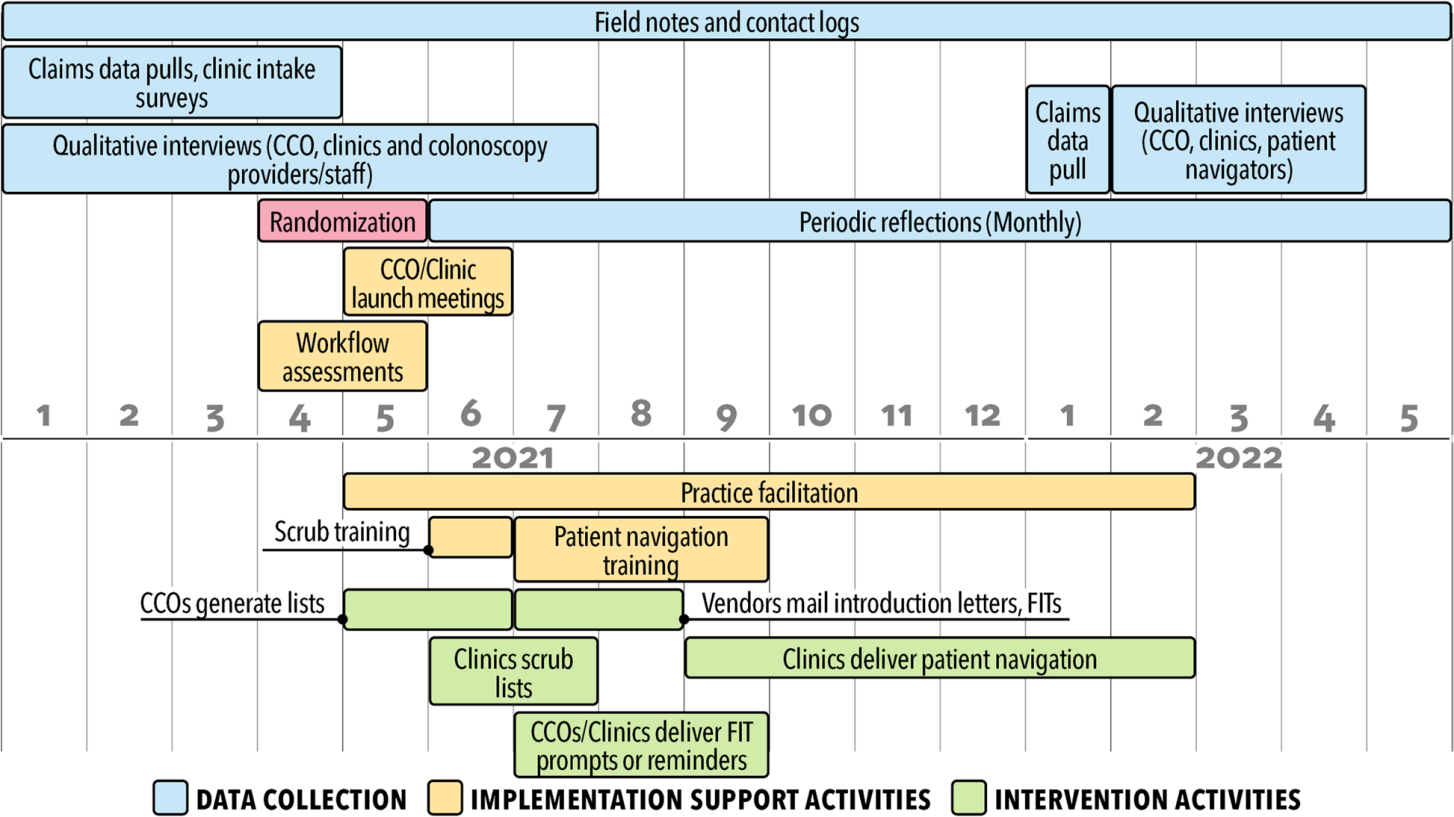
Timeline for first-year data collection and intervention delivery for SMARTER CRC study*. *In year 1, the program will be delivered to 15 clinic units allocated to the intervention; year 2 plans are to deliver the program to all participating clinic units (*n* = 29)

To realize aim 1, we will assess the implementation, effectiveness, and maintenance of the mailed FIT outreach and patient navigation program using a two-arm, parallel, cluster-randomized design conducted over the course of 2 years. Our mailed FIT outreach program uses a collaborative clinic-health plan-vendor model designed and pilot-tested by our team as part of the BeneFIT study [56] and adapted for rural regions [55, 56]. The patient navigation component uses the New Hampshire Colorectal Cancer Screening Program [61], with adaptations based on prior implementations by our study team and pilot-testing for rural clinics [44, 62]. The implementation of the multi-component program is supported by workflow mapping, formal training, and ongoing implementation support provided by practice facilitators and other members of the research team. Figure 1 displays the activities for the program.

##### Randomization

Beginning with an initial list of 33 recruited clinics, we created three small clusters of clinics (two pairs and one trio) within the same health system to serve as randomization units. These clinics had common staff or enrollees, lacked claims data at the level of individual clinics, or were too small to otherwise meet eligibility criteria. This resulted in 29 clinic units for randomization.

Units were randomized 1:1 into intervention and control groups. Randomization was stratified on health system designation (e.g., hospital-affiliated clinics or independent clinics) and performed by the project statistician in two batches in April 2021 (2 CCOs) and May 2021 (1 CCO), with timing based on data availability. Each batch consisted of five strata, with 2–6 clinic units within a stratum. The randomized allocation sequence was generated using STATA version 16 (College Station, TX). The study condition (intervention or usual care) was randomly assigned during a virtual ‘roll of dice’ meeting attended by health plan and clinic representatives, with the intervention group being offered the intervention during year 1 of the study (April 2021) and the control group being offered the intervention during year 2 of the study (April 2022). Fifteen units were assigned to intervention and 14 units were assigned to usual care. For practical reasons, the research team and the clinic staff were unblinded to randomization assignment.

##### Selection of eligible enrollees

On an annual basis, CCO staff use claims data to generate a list of eligible enrollees for each clinic assigned to the intervention; eligible enrollees are aged 50–75, enrolled in Medicaid or dually enrolled in Medicaid and Medicare, and due for CRC screening (no claims evidence of colonoscopy within past 10 years, flexible sigmoidoscopy or CT colonography within 5 years, FIT within the past year, or mt-sDNA in the past 3 years). The study team enters the list into a cloud-based database, REDCap (Fort Lauderdale, FL). Clinic staff then may use the list to conduct a “scrub” of the data to remove the names of any enrollee who, based on electronic health record (EHR) data, is not due for CRC screening, has life-limiting comorbid conditions, is otherwise ineligible for FIT testing, or has not established care at the clinic [55].

##### Intervention activities

**Mailed FIT outreach**

For the mailed FIT outreach component, scrubbed lists are sent to a vendor (PrintSync Inc. [Beaverton, Oregon], Koko Graphix [Klamath Falls, Oregon], or Home Access Health Corporation [Hoffman Estates, IL]) who delivers outreach to enrollees on the list. For EOCCO and CHA, the vendor mails introduction letters (tailored for rural clinics [32, 63, 64]) to all enrollees on the list, followed 1 week later by a FIT test, with instructions to return the test by mail to the clinic or the clinic’s designated laboratory for processing. Mailed with the FIT are a wordless FIT instruction sheet [33], a letter (also tailored for rural clinics), and a pre-addressed and postage-paid envelope. For CHA and PacificSource, the vendor also delivers a reminder (via text message or letter) about 2 weeks after the FIT mailing. The mailed FIT test is the same one-sample FIT test used in-clinic, when possible; tests used in clinic include OC-Auto® and OC-Light®S by PolyMedco (Cortland Manor, NY); Insure®ONE™ by Clinical Genomics (Edison, NJ); Hemosure® iFOB Test Kit by Hemosure, Inc. (Irwindale, CA); and Henry Schein OneStep+ by Henry Schein Inc. (Melville, NY)). If the test normally used by a clinic is not mailable or if the clinic cannot provide their normal FIT test for the mailing program, the OC-Auto® FIT is used. For PacificSource, the vendor mails and processes the FITs; that is, the vendor mails a single brand FIT test (OC-Auto®), which includes an introduction letter (the letter is not mailed separately), a pre-paid envelope for FIT return, and instructions to mail the kit to the vendor laboratory for processing.

Intervention clinic staff are encouraged to make a live outreach call to enrollees, either before or after the FIT mailing, in accordance with mailed FIT program best practices [45]. Phone calls and texts are tracked in the REDCap database. Consistent with usual care, enrollees whose FIT results are abnormal and who need a colonoscopy are referred to either internal surgical centers or colonoscopy providers (for some clinics affiliated with a hospital) or to external gastroenterology specialty care facilities (hospital or ambulatory surgical center) in the regional area of the clinic. These enrollees are contacted by clinic staff who are trained in patient navigation to initiate the patient navigation component of the program. CCO staff are also offered training to provide patient navigation for clinics that are unable to staff this component of the intervention.

**Patient navigation**

The adapted patient navigation program is phone-based and covers four topic areas (the original program consisted of 6 topic areas) [44, 61, 62]. The navigators are clinic staff or CCO staff trained by the research team. During the initial topic call, the navigator introduces the program, assesses barriers, and collaborates with the enrollees to resolve barriers (this can require multiple phone calls). The subsequent three calls are timed to the colonoscopy appointment, with a call delivered 1 week prior to the colonoscopy appointment focused on colonoscopy preparation, another call delivered the day before the colonoscopy to check-in with the patient, and a final call delivered 2 weeks following the procedure focused on assuring that the enrollee understands the results. Additionally, 2 weeks after laboratory results are returned, the care management team of one CCO (PacificSource) calls enrollees with abnormal results to encourage them to schedule a follow-up colonoscopy. Navigation activities and enrollee outcomes are tracked in the REDCap database and in each clinic’s EHR.

##### Implementation support activities

**Implementation facilitation**

Study activities will be primarily facilitated by the project manager, practice facilitators (who are members of the research team), and other study team members with relevant expertise (e.g., data analysis, patient navigation). Practice facilitation is increasingly used as a centralized and unifying strategy to build practice capacity [65, 66] and support implementation that is tailored to the local context and stakeholder needs [66–73]. Three practice facilitators from the Oregon Rural Practice-based Research Network serve as liaisons between the researchers, CCOs, and clinics. They are trained in practice facilitation, quality improvement, CRC screening outreach methods, and study-specific data collection methods. Practice facilitators maintain ongoing contact with the clinical teams (e.g., clinical champion, point of contact, and implementation teams) implementing the program. Ongoing contact includes helping to determine needed intervention adaptations, arranging attendance at trainings and delivering certain training elements, notifying clinic staff of key intervention steps (such as mailing events or data uploads), and providing ongoing implementation support. The facilitators also support re-training and re-engagement following clinical staff turnover or role changes. For SMARTER CRC, facilitation activities consist of formal workflow assessments and scheduled or ad hoc meetings with clinical teams by phone, video conferencing, or email. In addition, a monthly meeting is held among the research project team, CCO representatives, and staff from all intervention clinics involved in the project, including patient navigators. The purpose of this meeting is to provide overall project status updates, facilitate collaboration, make mid-course corrections, receive booster training, and identify needed adaptations to the intervention. While facilitation activities were predominantly planned for in-person delivery, we shifted to remote interactions due to COVID-19-related travel restrictions.

**CCO and clinic staff training**

Intervention training activities cover how to scrub patient lists for mailed FIT eligibility and how to conduct patient navigation. For the scrub training, two research team members (a project manager and research associate or practice facilitator) train clinical staff from each intervention clinic in how to review lists of enrollees and remove names of enrollees determined to be ineligible based on health record data (two training sessions were held to accommodate the roll-out timing of the different CCO FIT mailings) with supplemental follow-up to answer questions as needed. The scrub training lasts about 60 min, and a recorded version of the training is offered via a web link to participants for later viewing or to train new staff. In addition, a 5-h patient navigation training is provided; this training was adapted from the PRECISE study [44]. The patient navigation training includes a series of pre-recorded videos (total duration: 60 min) on the importance of CRC screening and follow-up and the role of patient navigation; two 90-min live interactive webinar sessions focused on personal and structural barriers to colonoscopy, effective messages to promote follow-up colonoscopy, and interactive role-playing; a 60-min live interactive webinar session on using the REDCap system to track patient navigation interactions; and an optional 60-min pre-recorded video session on motivational interviewing. The patient navigation training is offered to at least one staff member in each participating intervention clinic.

**Workflow assessments**

To understand current CRC screening and follow-up practices within a given clinic, practice facilitators conduct workflow assessments in collaboration with staff at clinics assigned to the intervention. Two workflow assessment meetings are held: The initial meeting primarily focuses on the mailed FIT component; the second meeting primarily focuses on the patient navigation component. During these meetings, practice facilitators create visual ‘swim lane’ diagrams using Lucidchart© (South Jordan, UT). As part of the facilitated workflow assessment, clinic staff describe their current CRC screening process, choose program components to implement (e.g., live call reminders for mailed FIT) and aspects of program implementation (e.g., minimum number of navigation call attempts before an enrollee is considered unreachable), and discuss any planned adaptations. Workflow diagrams are then reviewed with clinic staff and research team members and refined based on feedback. Based on the workflow, clinic staff assign leads for the mailed FIT and patient navigation components.

##### Evaluation

**Effectiveness**

We will evaluate intervention effectiveness by assessing whether enrollees in the intervention clinics are more likely to obtain any CRC screening compared to patients in the usual care clinics within 6 months of the date the enrollees were identified as eligible (claims list pull date) (Table 1). Additional effectiveness outcomes include receipt of FIT, time to FIT completion, and FIT result; receipt of screening colonoscopy; and among those with an abnormal FIT result, referral to follow-up colonoscopy, follow-up colonoscopy receipt, and time to colonoscopy. We also will assess colonoscopy outcomes (e.g., *n* adenomas, cancers detected) and referral to cancer care among those with cancer detected. To obtain data for outcomes unavailable in claims data (FIT positivity, etc.), practice facilitators will conduct chart audits in each participating clinic (at 1 year after the FIT mailing date). Primary analyses will rely on intention-to-treat; that is, enrollees will retain their unit at the time of randomization assignment irrespective of whether they received the mailed FIT and patient navigation intervention (analysis will retain enrollees who are removed during the scrub process) or transfer care to another randomization unit during follow-up. Because we do not anticipate having auxiliary variables that correlate greater than .50 with the outcome that would allow us to use multiple imputation [74, 75], we will handle missing outcome data for eligible enrollees who are lost to follow-up by assuming these enrollees did not complete any CRC screening. In addition, we will conduct a sensitivity analysis by dropping patients with missing outcome data because of CCO disenrollment (i.e., listwise deletion).

**Table 1:** Effectiveness and implementation outcomes for the SMARTER CRC study

Variable	Definition	Population
**Outcomes—effectiveness**		
Colorectal cancer screening completion—individual level (primary)	Receipt of any colorectal cancer screening (FIT, sDNA-FIT, colonoscopy, CT-colonography, sigmoidoscopy) within 6 months of the claims list pull date^b^ (binary)	Enrollees on claims lists (eligible enrollees)^a^
Colorectal cancer screening completion—clinic level	Clinic-level rates of receipt of any colorectal cancer screening (FIT, sDNA-FIT, colonoscopy, CT-colonography, sigmoidoscopy) within 6 months of the claims list pull date^b^ (proportion)	Enrollees on claims lists (eligible enrollees)^a^, aggregated by clinic
Time to CRC screening	Days from FIT mailing to screening completion, those who do not complete screening are censored at 12 months. Those who are lost to follow-up are censored on date of loss.	Enrollees on claims lists (eligible enrollees)^a^
FIT completion	FIT completed within 6 months of the claims list pull date^b^ (binary)	Enrollees on claims lists (eligible enrollees)^a^
Completion of screening colonoscopy	Colonoscopy completed within 6 months of the claims list pull date^b^ (binary)	Enrollees on claims lists (eligible enrollees)^a^
Fecal test result	Receipt of a normal/abnormal FIT test result (binary)	Eligible enrollees who completed a FIT within 6 months of the claims list pull date
Follow-up colonoscopy referral	Receipt of a colonoscopy referral within 6 months of the enrollee’s abnormal fecal test date (binary)	Eligible enrollees with an abnormal fecal test result
Follow-up colonoscopy completion	Receipt of a colonoscopy within 6 months of the enrollee’s abnormal fecal test date (binary)	Eligible enrollees with an abnormal fecal test result
Time to follow-up colonoscopy	Time from abnormal FIT test result to completed colonoscopy (time to event), those who do not complete a colonoscopy are censored at 6 months. Those who are lost to follow-up are censored on date of loss.	Eligible enrollees with an abnormal fecal test result
Colonoscopy outcomes	Detection of adenomas, advanced adenomas, or cancer (binary)	Eligible enrollees with a completed colonoscopy
Referral to cancer care	Receipt of referral to cancer care within 3 months of cancer diagnosis (binary)	Eligible enrollees with colorectal cancer detected
**Outcomes—implementation**		
Implementation	CCO- and clinic-level rates of program delivery, by core component (mailed FIT, patient navigation) and non-core components (clinic scrub, reminders delivered by clinics/CCOs); (proportion)	Eligible enrollees, by core and non-core intervention components, aggregated by clinic
Reach (enrollee level)	Receipt of the program, by component (mailed FIT sent to valid address, at least one patient navigation phone call received)	Eligible enrollees, by component (mailed FIT, patient navigation), aggregated by clinic
Adaptations to intervention at CCO and clinic levels, and adaptations to implementation strategies	CCO-clinic-research team level: adaptation, reason, type, who made decision to adapt	CCO staff, clinic staff, research staff
Implementation barriers and facilitators and contextual factors at CCO and clinic levels	Barriers and facilitators to implementation (qualitative); contextual factors	CCO staff, clinic staff
Reaction to the program/acceptability, at CCO, clinic, and colonoscopy provider/staff levels	Reactions to the intervention and implementation support (for clinics and CCOs), suggestions for improvement	CCO staff, clinic staff, colonoscopy provider and staff
**Outcome—maintenance**		
Maintenance at CCO, clinic, and enrollee levels	CCO/clinic level: implementation in year 2 (by component); enrollee level: CRC screening completion in year 2 (as appropriate)	CCO/clinic level: year 1 CCOs and intervention clinics that implemented the program; enrollee level: eligible enrollees who completed a FIT in year 1^c^
**Aim II: Scale-up**		
Adoption at the organizational and staff levels	Number of health plan, clinic, or community organization staff that participate in scale-up events, by event; proportion health plans, clinics, or community organizations that adopt the program; characteristics of adopters and non-adopters	Health plans, clinics, organizations that serve rural populations and were approached for participation
Implementation	Number of community organizations whose staff have facilitated health plans or clinics to deliver the program, and the number of health plans or clinics who have begun to implement the program, by component	Health plans, clinics, organizations that serve rural populations and were approached for participation
Adaptations	Adaptations made to the program; type, reason, who made decision to adapt	Health plans, clinics, organizations that adopt the program

^a^Enrollees on claims list are ages 50–75, and overdue for colorectal cancer screening, based on HEDIS criteria

^b^Lists pull dates generally vary by CCO

^c^Enrollees who completed colonoscopy, sigmoidoscopy, CT colonography, or FIT-DNA in year 1 will be excluded

### Statistical analysis

We will use hierarchical generalized linear modeling to account for clustering of enrollees within clinics in our assessment of intervention effectiveness (clinic level), implementation, and maintenance (CCO, clinic, and enrollee levels) [76]. Because the primary outcome is binary (i.e., any CRC screening, yes/no), we will use a model with a logit link and binomial distribution (i.e., multi-level logistic regression). The independent variable will be arm (dummy-coded) with usual care as the reference group. Clinic (or clinic cluster) will be modeled as a random effect. Odds ratios >1 support the hypothesis that the adapted mailed FIT program with patient navigation support increases the likelihood of an enrollee obtaining any CRC screening compared to usual care. A similar framework will be used for the other binary outcome variables (e.g., receipt of FIT, receipt of screening colonoscopy, referral to follow-up colonoscopy, receipt of follow-up colonoscopy). To compare time to FIT and time to follow-up colonoscopy, we will use Cox proportional hazards regression with shared frailty to account for the clustering of patients nested within clinics.

**Sample size and power**

Assuming a balanced, two-arm, cluster-randomized trial, 106 patients per clinic, an intra-class correlation (ICC) of .03, and a pre-implementation (baseline) CRC screening rate of 44.6%, we will have 79.5% power to detect a clinically meaningful change in screening rates as small as 10% in the intervention group (an increase to 54.6%) following program implementation using a two-tailed alpha level of .05. Pre-implementation (baseline) CRC screening rates were determined by reviewing clinic-level data from EOCCO and excluding clinics with a CRC screening rate above 60%. The minimum detectable effect size (MDES) to achieve 80% power is 10.1%, using the same assumptions. For ICC values ranging between .01 and .05, power ranges from 97.7 to 62.1%, and MDES to achieve 80% power range from 7.1 to 12.3%, indicating moderate sensitivity to the value of the ICC [77, 78]. Power calculations were determined using PASS 15 [79].

**Implementation outcomes**

We will evaluate intervention implementation by assessing the extent to which intervention clinics deliver the program, by component (for mailed FIT: within 6 months of the date the enrollees were identified as eligible (claims list pull date); for patient navigation within 6 months of the abnormal FIT result date). Additional implementation outcomes include implementation of non-core components (e.g., list scrubbing at the clinic level, mailed FIT reminders at the CCO and clinic levels), reach, acceptability (measured at the CCO, clinic, colonoscopy provider/staff, and enrollee levels), barriers and facilitators, and program adaptations (desired and executed). We will also track the number of CCO and clinic staff who participate in the scrub training and patient navigation training. Implementation will be assessed using mixed methods, that is, data in REDCap (intervention activities) and vendor data (intervention activities and screening events) as well as findings from a baseline intake survey, one-on-one qualitative interviews (conducted before and after implementation), field notes taken by practice facilitators, and periodic reflections among members of the study team.

**Clinic intake survey**

Prior to randomization, a clinic intake survey was completed by all participating clinics. The clinic intake survey was developed using common data elements from the ACCSIS consortium. The survey asks about what CRC screening tests the clinic uses, CRC screening rates, rates of follow-up colonoscopy, and characteristics of the health system to which the clinic belongs. The survey was managed using REDCap; links to the survey were distributed by email to the primary point of contact in all recruited clinics (intervention and usual care); practice facilitators followed up with clinic staff, as needed, to obtain answers to any skipped questions and to document survey questions for which the answer could not be easily obtained.

**Qualitative interviews**

Semi-structured qualitative interviews among CCO leaders in quality improvement roles (*n* ~ 3), clinic staff (clinic managers, quality improvement staff, physicians; *n* ~ 30), and clinicians and staff from colonoscopy facilities (clinic managers, scheduling/referral staff, billing specialist, or clinicians; *n* ~ 20) are conducted prior to the FIT mailing and after implementation. These interviews will be conducted in intervention and usual care clinics. Practice facilitators will conduct informal qualitative interviews with enrolled clinics 6–9 months from the year 1 FIT mailing for the purpose of informing year 2 implementation. Interviews with clinic and CCO staff seek to understand contextual factors, barriers and facilitators to implementation and maintenance, program acceptability and adaptations (both desired and executed), and unanticipated consequences (positive or negative). Interviews with clinicians performing colonoscopy and their staff seek to understand their awareness of and reaction to the program. Interviews will also be conducted with participating CCOs and clinics 9 months following the post-implementation interview to assess maintenance of the intervention. Interviews are conducted via videoconference and generally last 30–60 min. All qualitative interviews will be digitally recorded, professionally transcribed, uploaded to ATLAS.ti, and then analyzed by the research team using an immersion crystallization approach to identify salient findings [80]. Findings from the interviews will inform program scale-up activities in aim 2 and will be used to develop content for study dissemination materials.

**Periodic reflections and field notes**

We will conduct monthly periodic reflection meetings with the practice facilitators and other members of the research team to track adaptations (to the intervention and to implementation strategies) and to understand barriers, facilitators, and contextual factors at the clinic and CCO levels [81]. In addition, communication logs documenting interactions with CCOs and clinics will be maintained by each member of the research team, and clinic staff will enter notes into the REDCap database. Field notes and transcripts of periodic reflection sessions will be analyzed according to the immersion-crystallization approach on a quarterly basis throughout implementation [80].

**Implementation evaluation**

To guide our evaluation of the implementation process, we will use elements of the Proctor Implementation Framework and RE-AIM [82, 83]. We will assess program implementation at the CCO and clinic levels using process measures (e.g., number of kits mailed/number anticipated, number eligible enrollees who receive any navigation). Reach is defined as the number of enrollees who received the intervention/number eligible enrollees, by component (mailed FIT or patient navigation). We will aggregate and report reach at the level of the clinic and CCO. We will also assess the characteristics of those reached and not reached. We will track adaptations to the program and implementation support using components of the FRAME framework for intervention adaptations and the FRAME-IS framework for implementation strategy adaptations [84, 85], focusing on adaptation type and the reason the adaptation was made. We will build on prior application of the FRAME framework by our team [86].

**Maintenance**

We will assess maintenance at the CCO, clinic, and enrollee levels. Maintenance will be defined as the proportion of CCOs and clinics that implemented the program in year 1 that also implemented it in year 2. For those CCOs and clinics that continue the program in the second year, we will descriptively compare the screening rates at year 2 to that of year 1. At the enrollee level, maintenance is defined as the proportion of enrollees who completed a FIT in year 1 also completed one in year 2 (with censoring for those who received a colonoscopy and thus are ineligible for FIT in year 2).

#### Aim 2: scale up the program to additional clinics serving rural and underserved patients using webinars, train-the-trainer workshops, and collaborative learning approaches

**Fig. 2:**
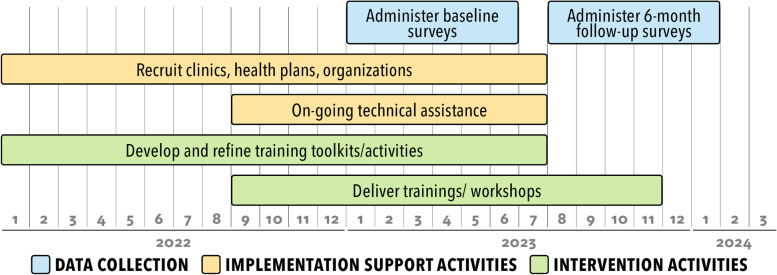
Timeline for the scale-up of the SMARTER CRC program

We will work with a national advisory board to develop a plan to scale up the program and disseminate training products to a national audience. Findings from the cluster-randomized study will be used to inform scale-up planning. Partnering regional and national organizations will receive a small stipend to participate in program training sessions to facilitate outreach and implementation of the mailed FIT and patient navigation intervention in 5–10 clinics they serve and to support program evaluation and tracking activities. The plan will likely include training sessions on mailed FIT outreach, patient navigation, and practice facilitation delivered to health systems, as well as to technical assistance and advocacy-organizations, using webinars, train-the-trainer workshops, and learning collaboratives informed by the Extension for Community Healthcare Outcomes (ECHO) format (Fig. 2). The 5-h multi-modal patient navigation training program developed for the main trial will be adapted, as needed, and delivered to support asynchronous and synchronous interactive learning. Two-day train-the-trainer workshops will be offered for individuals who provide technical assistance or quality improvement support. The project practice facilitators will be available by phone or email for organization staff who need real-time assistance implementing program components.

As part of the registration process for webinars, training workshops, and learning collaboratives, participants will be asked to complete a brief (15-min) survey. Survey questions will assess adoption of the program, program adaptations implemented or planned, and current clinical practices related to CRC screening and follow-up (e.g., current CRC screening practices and policies, screening test use/emphasis, and lab and vendor relationships, community resources). Participants who represent community organizations will be asked about their organization’s current CRC screening and follow-up facilitation activities and future plans. For all participants, we will gather demographic information (e.g., age, sex), professional role, affiliated organization name and location, and email address. We will ask permission to recontact participants in 6 months for a follow-up survey. Participants of multiple SMARTER CRC training events will only be asked to complete a single baseline survey.

Six months following the administration of the baseline survey, project staff will email respondents an invitation to complete a follow-up survey. This survey will ask respondents about their clinical practices related to CRC screening and follow-up, the extent to which implementation of the SMARTER CRC program is being planned or executed, planned or implemented adaptations to the program, and their participation in SMARTER CRC trainings or workshops.

Our plan to evaluate the scale-up trial will be developed with guidance from the national advisory board. We will track the number of health plan, clinic, and community organization staff who attend our train-the-trainer workshops, mailed-FIT workshops, and patient navigation workshops, ECHO-informed learning forums, and project-specific training events. We will report the number of individuals trained on each component by role (e.g., clinic staff, community organization representative, other). Using 6-month follow-up survey data, we will assess the number of community organizations whose staff have facilitated health plans or clinics to deliver the program and the number of health plans or clinics who have begun to implement each component of the program. Among those who have implemented the program, we will collect and report information about adaptations to the program and the reasons adaptations were made. We will assess determinants of dissemination success, using methods from systems science or configurational comparative methods [87, 88]. We will maintain project materials on a website and track the number of times the website is accessed and the number of times our implementation guide and templates are downloaded.

## Discussion

SMARTER CRC is a pragmatic study that will test the implementation, effectiveness, and scale-up of a multi-component mailed FIT and patient navigation program delivered to rural Medicaid and dual Medicaid-Medicare enrollees using a partnership among clinics, health plans, and vendors. We will test the program in three Medicaid health plans and 29 rural clinic units, then scale up the program by delivering tailored trainings and implementation support to health systems and organizations, assessing program adoption and adaptations. Our findings may have a profound impact on how to broadly scale evidence-based CRC screening programs in rural and frontier settings and may be particularly valuable in the context of COVID-19-related care disruptions and recent changes to the US Preventive Services Task Force CRC screening guidelines.

## Data Availability

Data sharing is not applicable to this article as no datasets were generated or analyzed during the current study.

## Abbreviations

SMARTER CRC: Screening More patients for CRC through Adapting and Refining Targeted Evidence-based Interventions in Rural settings
FIT: Fecal immunochemical test
CRC: Colorectal cancer
CCO: Coordinated Care Organization
EOCCO: Eastern Oregon Coordinated Care Organization
CHA: Cascade Health Alliance
ICC: Intra-class correlation coefficient
MDES: Minimum detectable effect size
ECHO: Extension for Community Healthcare Outcomes
